# *In vivo* cardiovascular magnetic resonance of 2D vessel wall diffusion anisotropy in carotid arteries

**DOI:** 10.1186/s12968-016-0304-8

**Published:** 2016-11-23

**Authors:** Peter Opriessnig, Harald Mangge, Rudolf Stollberger, Hannes Deutschmann, Gernot Reishofer

**Affiliations:** 1 Clinical Institute for Medical and Chemical Laboratory Diagnosis, Medical University of Graz, Auenbruggerplatz 15, A-8036 Graz, Austria; 2 Institute of Medical Engineering, Graz University of Technology, Stremayrgasse 16/III, A-8010 Graz, Austria; 3 Department of Radiology, Division of Neuroradiology, Vascular and Interventional Radiology, Medical University of Graz, Auenbruggerplatz 9, A-8036 Graz, Austria

**Keywords:** Cardiovascular magnetic resonance, Diffusion tensor imaging, Cardiovascular diseases, Atherosclerosis

## Abstract

**Background:**

Diffusion weighted (DW) cardiovascular magnetic resonance (CMR) has shown great potential to discriminate between healthy and diseased vessel tissue by evaluating the apparent diffusion coefficient (ADC) along the arterial axis. Recently, ex vivo studies on porcine arteries utilizing diffusion tensor imaging (DTI) revealed a circumferential fiber orientation rather than an organization in axial direction, suggesting dominant diffusion perpendicular to the slice direction. In the present study, we propose a method to access tangential and radial diffusion of carotids in vivo by utilizing a pulse sequence that enables high resolution DW imaging in combination with a two-dimensional (2D) diffusion gradient direction sampling scheme perpendicular to the longitudinal axis of the artery.

**Methods:**

High resolution DTI of 12 healthy male volunteers (age: 25–60 years) was performed on one selected axial slice using a read-out segmented EPI (rs-EPI) sequence on a 3T MR scanner.

**Results:**

It was found consistently for all 12 volunteers, that the tangential component as the principle direction of diffusion. Mean vessel wall fractional anisotropy (FA) values ranged from 0.7 for the youngest to 0.56 for the oldest participant. Linear regression analysis between the FA values and volunteers age revealed a highly significant (*P* < 0.01) linear relationship with an adjusted R^2^ of 0.52. In addition, a linear trend (*P* < 0.1) could be observed between radial diffusivity (RD) and age.

**Conclusion:**

These results point to FA being a sensitive parameter able to capture changes in the vascular architecture with age. In detail, the data demonstrate a decrease in FA with advancing age indicating possible alterations of tissue microstructural integrity. Moreover, analyzing 2D diffusion tensor directions is sufficient and applicable in a clinical setup concerning the overall scan time.

## Background

Cardiovascular diseases (CVDs) remain the global killer number one. Estimations from the world health organization (WHO) revealed, that about one third of global deaths were attributable to this life threatening diseases in 2012 [1]. Of these events, three quarters are associated with stroke and coronary heart disease. The most severe pathological condition is atherosclerosis (AS), affecting blood and heart vessels. AS is a chronic immune-mediated inflammatory state of large and medium sized arteries that manifests in the first decade of life, with silent progression until a clinical event takes place [2]. The detection and diagnosis of AS at an early stage plays a key role in health care allowing for personalized interventions adjusted to the needs of patients.

Imaging modalities, such as ultrasound, computed tomography (CT), cardiovascular magnetic resonance (CMR) can assist in the detection and classification of morphological pathologies. However, these imaging techniques are applied mainly to determine the degree of luminal narrowing, which is an indispensable information, but a limited biomarker to determine the true impact on future cardiovascular events [3]. A more advanced imaging technique is multi-contrast CMR which became a well-recognized tool to study the complex composition of atherosclerotic plaques [4, 5]. The intrinsic soft tissue contrast and high spatial resolution makes CMR a well suited tool to delineate key constituents of plaque vulnerability.

Recently, diffusion-weighted (DW) CMR has shown great potential to discriminate between normal and pathological vessel tissue by evaluating the apparent diffusion coefficient (ADC) [6–8] for a certain vascular segment. Given the fact that the spatial resolution is limited in DWI as a consequence of a dramatically increase in echo time when using conventional diffusion measurements, the diffusion perpendicular to an artery’s axis has never been imaged in vivo so far. The specific architecture of vessels allows for the investigation of diffusion anisotropy in the vessel wall by diffusion tensor imaging (DTI), which has already been shown in ex vivo studies where the fibrous structure of porcine aortas has successfully been visualized [9, 10].

Two major constraints prevent the application of DTI to visualize diffusion in the carotid artery wall in vivo. Firstly, the low spatial resolution when using a conventional single-shot EPI (ss-EPI) and a significant loss of SNR at higher spatial resolution as a result of the prolonged echo time. Secondly, the scan time that dramatically increases with the number of diffusion sensitizing gradient directions.

In this work we propose a method to overcome these limitations in order to measure tangential and radial diffusion, evaluated from a two-dimensional diffusion tensor, in the carotid artery wall in vivo. High spatial resolution was achieved by utilizing a read-out segmented EPI (rs-EPI) pulse sequence [11]. Due to the specific properties of this pulse-sequence, sufficient SNR can be gained to allow for DTI analysis. Clinically acceptable scan time was assured by using a novel gradient direction scheme. Motivated by results from ex vivo DTI studies [9, 10] that revealed a tangential orientated principle diffusion direction and further supported by our ex vivo measurements, the usually used three-dimensional gradient direction scheme was reduced to a two-dimensional acquisition scheme that significantly decreased the number of needed gradient directions.

In this proof of concept we demonstrate the feasibility of measuring diffusion in the vessel wall of carotid arteries in vivo in a non-destructive and non-invasive fashion and provide initial results from 12 healthy male volunteers.

## Methods

### Two-dimensional diffusion acquisition scheme

**Fig. 1:**
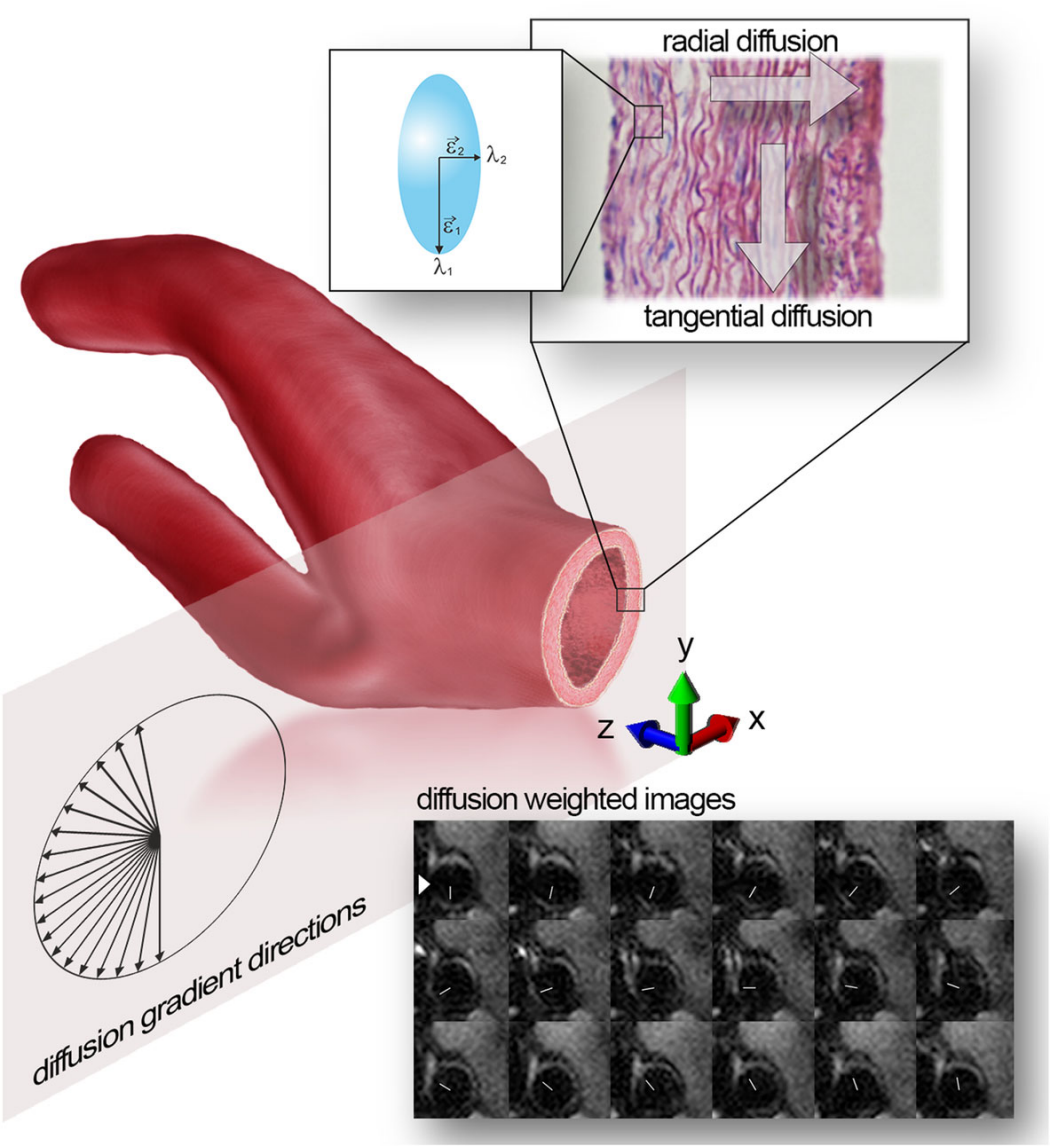
Gradient direction scheme to measure tangential and radial diffusion. *Top*, The concept of tangential and radial diffusion is outlined as well as the 2D ellipsoid with eigenvalues (λ) and eigenvectors (ε). *Middle*, The sketch outlines the anatomical region of interest (carotid artery) and the orientation of the 18 diffusion gradient directions on a hemicycle within a plane perpendicular to the longitudinal axis of the carotid artery (*black arrows*). *Bottom*, Representative in vivo DWI from a carotid artery at a b-value of 600 s/mm^2^ for the 18 diffusion directions (*white headless arrows*)

The basic idea to reduce the amount of diffusion sensitizing gradients is the reduction of a three-dimensional diffusion measurement to a two-dimensional diffusion measurement perpendicular to the artery’s axis (Fig. 1). This strategy is justified by the work from Flamini et al. [10] revealing a tangential fiber orientation that indicates the principle direction of diffusion. Given the fact that diffusion in z-axis is low compared to tangential and radial diffusion, this simplification provides the basis for in vivo measurements due to the significant reduction of diffusion sensitizing gradients needed for DTI. To demonstrate the validity of our approach 3D and 2D diffusion measurements on a porcine aorta were performed.

### Preliminary work on porcine aorta

Ex vivo *DWI* of a slaughterhouse harvested porcine aorta was performed on one selected axial slice using rs-EPI and both a three-dimensional gradient direction schema with 30 directions (Multi Directional Diffusion Weighting, MDDW; acquisition time = ~50 min) and a two-dimensional schema with 18 directions (acquisition time = ~30 min). Sequence parameters were as follows: FOV = 111 × 111 mm^2^, matrix = 308 × 308, slice thickness = 10 mm, TR = 2500 ms, TE = 81 ms, GRAPPA = 2, number of readout segments = 31, b-values = 0 and 1000 s/mm^2^. To extract angle distributions of the principle 3D-DTI tangential vector with respect to the longitudinal axis of the artery Eq. (1) was used.

1 $$ \uptheta =\frac{\pi }{2}- \arccos \frac{z}{\sqrt{x^2+{y}^2+{z}^2}} $$

To optimize imaging conditions, ex vivo specimens were immersed in a fluid (1,1,2-Trichloro-1,2,2-trifluoroethane, Genetron® 113, Sigma-Aldrich) having no MR signal and to minimize magnetic susceptibility effects at air-tissue boundaries [12, 13].

### In vivo MR imaging

The 2D diffusion acquisition scheme was used for in vivo measurements requiring that the diffusion sensitizing directions, lying within a plane in space, are oriented perpendicular to the artery’s axis. This was achieved by defining the diffusion directions in the (p,r,s) coordinate system that is linked to the orientation of the field of view.

*High resolution diffusion weighted imaging* (DW) of 12 healthy male volunteers (25–60 years) was performed on one selected axial slice using rs-EPI on a 3T whole body MR scanner (Prisma, Siemens Medical Solutions, Erlangen, Germany) with a 2 × 4 channel multifunctional coil (NORAS MRI products GmbH, Germany) and FOV = 189 × 189 mm^2^, matrix = 346 × 346, slice thickness = 10 mm, TR = 2RR intervals, trigger delay = 50 ms to acquire images in the diastolic phase, TE = 93 ms, GRAPPA = 2, number of readout segments = 9, b-values = 0, 200, 400, 600 s/mm^2^, acquisition time = ~12 min. Eighteen gradient directions were defined on a hemicycle within a plane oriented perpendicular to the longitudinal axis of the carotid artery. To minimize artifacts, introduced by pulsatile vessel motion, peripheral pulse-triggering was performed using the systems’ internal physiological monitoring unit. To avoid chemical shift artifacts caused by peri-adventitial fat, a spectral fat saturation pulse was applied. A series of *two-dimensional (2D) angiographic axial vessel scout* images (TurboFLASH: TR = 500 ms, TI = 280 ms, TE = 4.65 ms, flip angle = 15 °, FOV = 150 × 150 mm^2^, matrix = 256 × 102, slice thickness = 5 mm, acquisition time = ~1 min) preceded the main task to locate the carotids and orient the slice perpendicular to the long axis of the artery. Measured DWI data with an in-plane resolution of 0.55 × 0.55 mm^2^ were interpolated to 0.2 × 0.2 mm^2^ using zero-filling.

*Registration* of the individual DW images was achieved by applying publicly available elastix software [14, 15]. In detail, registration of the individual DW images was applied to the b0 image. The registration process included a rigid registration (in-plane translation only) to correct for motion introduced by the relaxing back muscles during the investigation. In a next step the wall of the individual DW images was manually segmented to prevent misregistration to the dominant spine/neck structures. As a final processing step in order to correct for distortions introduced by more complex motion (eddy currents, breathing, swallowing and residual vessel motion) a B-spline transform was applied to register the delineated wall DW images [16, 17].

The study was approved by the institutional review board (IRB) as well as followed Declaration of Helsinki recommendations and informed consent was signed by each participant prior to the MR measurements.

### Reproducibility of in vivo FA measurements

To test the robustness of repeated FA measurements using the above introduced in vivo high resolution DW sequence, the carotid artery of four male volunteers was repeatedly (in total four visits) measured and the coefficient of variation (CV), defined as the ratio of the standard deviation (σ) to the mean (x̅), was calculated.

### Multiple b-value selection for the in vivo DWI imaging experiment

The afore mentioned in vivo high resolution diffusion weighted imaging (DWI) parameter setup was used to investigate optimal b-values on one selected axial carotid slice of a male volunteer. With all other sequence parameters held constant, the TE was set to 97 ms in order to allow for measurements with increasing b-values from 0 to 1000 s/mm^2^ (in steps of 200 s/mm^2^) for a certain diffusion gradient direction perpendicular to the slice direction.

### Statistical analysis

To investigate the relationship between fractional anisotropy (FA), radial diffusivity (RD) and primary eigenvalue (λ_1_) data with volunteer’s age a *linear regression analysis* was used to estimate parameters (coefficient of determination (R^2^), slope and intercept) and assess linearity. In addition, 95 % confidence and prediction intervals for the linear regression were calculated. An *F-test* for linear models was employed to compare variances and a probability value of *P* < 0.05 was considered as significant. Normal distribution was tested based on *Shapiro-Wilk Normality Test*. All analyses were performed using freely available R-project software [18].

### Diffusion tensor mathematics, FA, RD, MD and primary eigenvalue calculation

The 3D diffusion tensor described by a symmetric 3 × 3 matrix and its derived quantities can easily be reduced to the 2D case where the tensor is defined by a 2 × 2 matrix. Mathematical aspects are discussed in the following and are detailed described by Kingsley et al. [19–21]. A Levenberg-Marquardt nonlinear least squares fitting algorithm was applied based on Eq. (2) to calculate diffusion coefficients (D, measured apparent diffusion coefficient) for every voxel for all measured gradient directions. Attenuation of the MR signal S(b) is introduced by varying the b value (200, 400, 600 s/mm^2^) sensitizing it to water diffusion. S_0_ represents the reference signal without diffusion weighting (no diffusion gradient).

2 $$ \mathrm{S}\left(\mathrm{b}\right)={\mathrm{S}}_0{\mathrm{e}}^{-\mathrm{b}\mathrm{D}} $$

The 2D gradient direction sampling scheme enables the evaluation of the diffusion tensor **d** by solving Eq. (3):

3 $$ \mathbf{Y}=\mathbf{H}\mathbf{d} $$

where **Y** represents the calculated apparent diffusion coefficients (18 element row vector), **H** is derived from the normalized gradient components Eq. (4) and **d** is the tensor given as a three-element column vector Eq. (5).

4 $$ \mathbf{H}=\left(\begin{array}{ccc}\hfill {g}_{x_1}^2\hfill & \hfill {g}_{y_1}^2\hfill & \hfill 2{g}_{x_1}{g}_{y_1}\hfill \\ {}\hfill \vdots \hfill & \hfill \vdots \hfill & \hfill \vdots \hfill \\ {}\hfill {g}_{x_{18}}^2\hfill & \hfill {g}_{y_{18}}^2\hfill & \hfill 2{g}_{x_{18}}{g}_{y_{18}}\hfill \end{array}\right) $$

5 $$ \mathbf{d}={\left[{\mathrm{D}}_{\mathrm{xx}}\ {\mathrm{D}}_{\mathrm{yy}}\ {\mathrm{D}}_{\mathrm{xy}}\right]}^{\mathrm{T}} $$

From tensor **d** corresponding eigenvalues (λ) and eigenvectors (ε) can be calculated on a pixel by pixel basis using eigenvalue decomposition. To study tangential and radial diffusion of the vessel wall 2D FA values were obtained using Eq. (6).

6 $$ \mathrm{F}\mathrm{A}=\frac{\left({\uplambda}_1-{\uplambda}_2\right)}{{\left({\uplambda}_1^2+{\uplambda}_2^2\right)}^{\frac{1}{2}}} $$

FA values close to zero mean no directed diffusion whereas anisotropy is given if one of the eigenvalues will be higher than the other. Calculation included the primary eigenvalue (λ_1_), RD (λ_2_ for the two-dimensional tensor) and the mean diffusivity (MD) which is the average of the eigenvalues.

## Results

### In vivo vessel wall diffusion weighted imaging

**Fig. 2:**
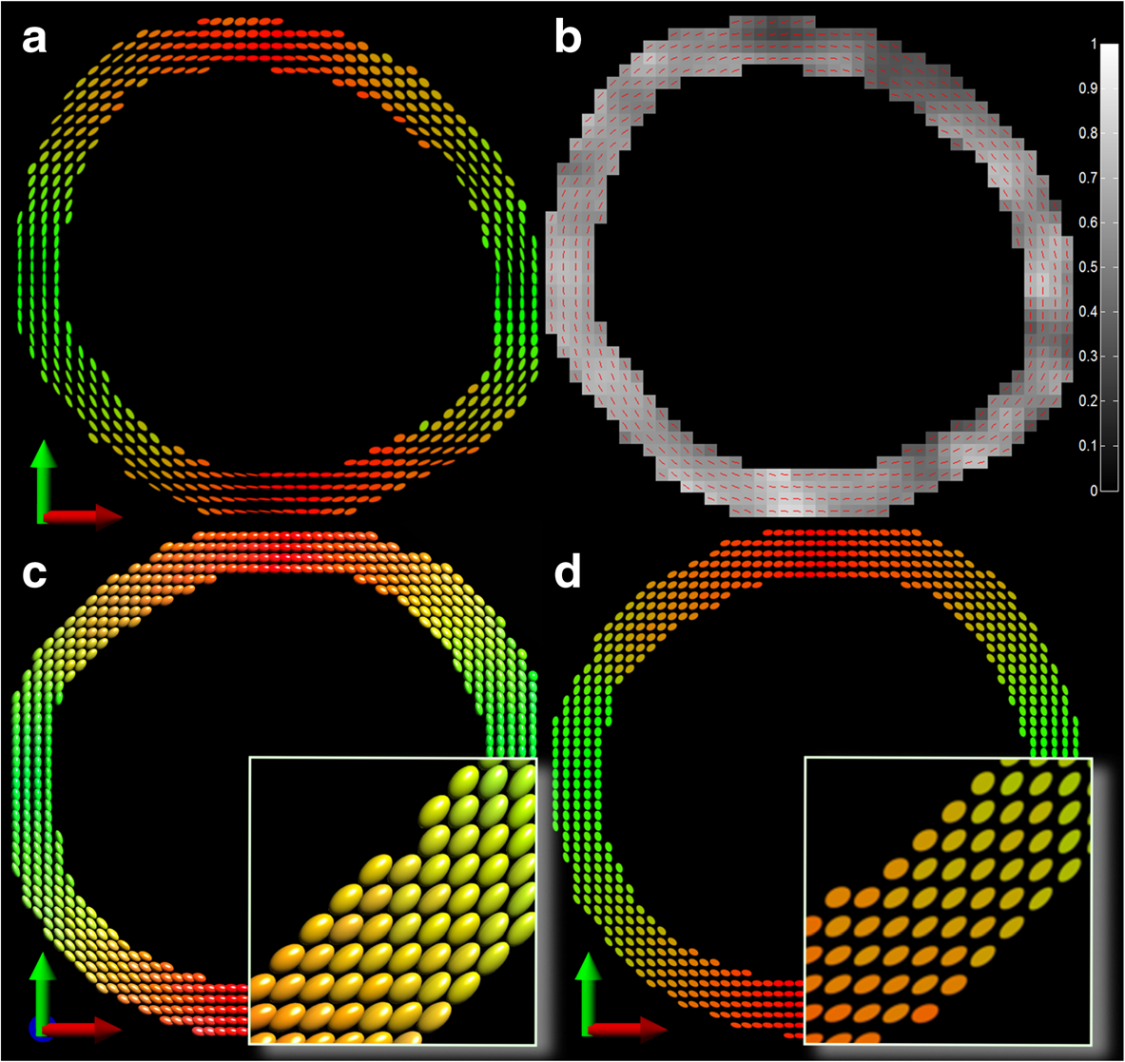
Color coded vector images of the principle diffusion tensor direction and FA map. **a** Representative RG vector image illustrating the diffusion tensor directions as 2D ellipsoids. **b** Corresponding FA map with superimposed direction of the principle DTI vectors by *red lines*. **c** RGB vector image based on the 3D-DTI rs-EPI with 30 gradient directions representing diffusion tensor directions as 3D ellipsoids of a porcine aorta. **d** RG vector image based on the 2D-DTI rs-EPI with 18 gradient directions representing diffusion tensor directions as 2D ellipsoids of the same porcine aorta

Table 1 summarizes the calculated mean diffusion components as well as the mean diffusivity (MD) and FA for the high resolution 2D-DTI in vivo case of the 12 male volunteers. The tangential and radial diffusion components correspond to the eigenvalues λ_1_ and λ_2_, respectively. Figure 1 outlines the anatomical region of interest (carotid artery) and the orientation of the diffusion gradient directions on a hemicycle within a plane perpendicular to the longitudinal axis of the carotid artery. The concept of tangential- and radial diffusion as well as the two-dimensional diffusion ellipsoid with eigenvalues (λ) and eigenvectors (ε) is delineated. In addition, Fig. 1 shows representative in vivo DWI from the carotid artery at a b-value of 600 s/mm^2^ for the predefined set of diffusion directions (headless white arrows). It can be observed that the signal is suppressed in the direction of the diffusion sensitizing gradient, marked by the white arrowhead. Calculating the 2D diffusion tensor revealed consistently for all 12 volunteers that the largest eigenvalue is oriented tangential to the vessel wall while the second component therefore shows a radial orientation. This fact provides clear evidence for the tangential direction as the principle contribution for diffusion within the vessel wall as shown in Fig. 2a. Based on the eigenvalues a two-dimensional fractional anisotropy map (FA) was generated (Fig. 2b). Additionally overlain is the direction of the principal DTI vectors denoted by the red lines.

**Table 1:** Volunteer Demographics and mean diffusion components of the in vivo high resolution 2D-DTI case

Demographic		Diffusion tensor			
Volunteer	Age	Tangential (λ_1_)	Radial (λ_2_)	MD	FA
male	yr	mm^2^/s	mm^2^/s	mm^2^/s	
V1	27	1.59 ± 0.52	0.44 ± 0.32	1.02 ± 0.36	0.697 ± 0.192
V2	29	1.84 ± 0.47	0.55 ± 0.22	1.19 ± 0.28	0.659 ± 0.147
V3	32	1.85 ± 0.69	0.64 ± 0.36	1.24 ± 0.50	0.636 ± 0.151
V4	33	2.29 ± 0.80	0.71 ± 0.30	1.50 ± 0.50	0.653 ± 0.118
V5	33	3.61 ± 2.60	1.14 ± 0.52	2.38 ± 1.49	0.612 ± 0.131
V6	34	3.10 ± 1.07	1.11 ± 0.44	2.11 ± 0.67	0.592 ± 0.145
V7	37	2.05 ± 0.77	0.74 ± 0.43	1.40 ± 0.54	0.604 ± 0.180
V8	41	2.31 ± 0.63	1.04 ± 0.39	1.68 ± 0.44	0.500 ± 0.148
V9	44	2.56 ± 0.65	0.99 ± 0.48	1.78 ± 0.50	0.581 ± 0.181
V10	45	2.34 ± 0.79	0.91 ± 0.42	1.63 ± 0.55	0.571 ± 0.165
V11	47	2.58 ± 0.66	1.09 ± 0.41	1.84 ± 0.46	0.527 ± 0.163
V12	57	2.22 ± 0.54	0.88 ± 0.29	1.55 ± 0.38	0.563 ± 0.118
		x1e-3	x1e-3	x1e-3	

Data are reported as x̅ ± σ

### Robustness of in vivo FA measurements

Table 2 summarizes repeated measurements (#1 to #4) of individual mean FA values of four male volunteers (R1 to R2). The robustness of the measurement is demonstrated by the coefficient of variation (CV) with values ranging from 2.5 to 5.4 %.

**Table 2:** Repeatability of mean FA measurements tested on four male volunteers

Volunteer				
	R1	R2	R3	R4
#1	0.525	0.551	0.592	0.535
#2	0.562	0.585	0.604	0.540
#3	0.563	0.614	0.607	0.566
#4	0.597	0.617	0.628	0.602
σ	0.029	0.031	0.015	0.031
x̅	0.562	0.592	0.608	0.561
CV	5.3 %	5.2 %	2.5 %	5.4 %

### 3D and 2D diffusion comparison in porcine

To confirm the tangential diffusion as the direction of the largest eigenvalue ex vivo high resolution 3D-DTI and 2D-DTI of a slaughterhouse harvested porcine aorta was performed using rs-EPI. Figure 2c and d represents the 3D diffusion tensor as ellipsoids in a three- dimensional color space (RGB), and the 2D diffusion tensor in a two-dimensional color space (RG), respectively. Calculating the angle between the principle diffusion direction and the artery’s axis for the 3D measurement, the mean value of 1.8 ± 7.2 ° confirms the dominant orientation of diffusion perpendicular to the artery’s axis. The low diffusion in z-direction justifies the 2D diffusion measurement perpendicular to the artery’s axis allowing for in vivo measurements in a clinically feasible time due to the lower number of diffusion sensitizing gradient directions. In addition, Table 3 summarizes the calculated mean diffusion components for the high resolution 2D-DTI (tangential and radial) and 3D-DTI (tangential, radial and longitudinal) ex vivo case of the slaughterhouse porcine aorta. For the 2D case the diffusion components correspond to the eigenvalues λ_1_ and λ_2_. For the 3D case the tangential component corresponds to the largest eigenvalue (λ_1_). The radial and longitudinal components can either be λ_2_ or λ_3_ because both eigenvalues are in a comparable range of magnitude (Fig. 2c).

**Table 3:** Mean diffusion components of the ex vivo high resolution 2D-DTI (top) and 3D-DTI (bottom) case

2D				
tangential (λ_1_)	radial (λ_2_)	MD	FA	
mm^2^/s	mm^2^/s	mm^2^/s		
1.10 ± 0.08	0.65 ± 0.10	0.88 ± 0.08	0.36 ± 0.07	
x1e-3	x1e-3	x1e-3		
3D				
tangential (λ_1_)	radial	longitudinal	MD	FA
mm^2^/s	mm^2^/s	mm^2^/s	mm^2^/s	
1.07 ± 0.06	0.64 ± 0.10	0.62 ± 0.07	0.78 ± 0.06	0.32 ± 0.04
x1e-3	x1e-3	x1e-3	x1e-3	

Data are reported as x̅ ± σ

### b-value estimation

**Fig. 3:**
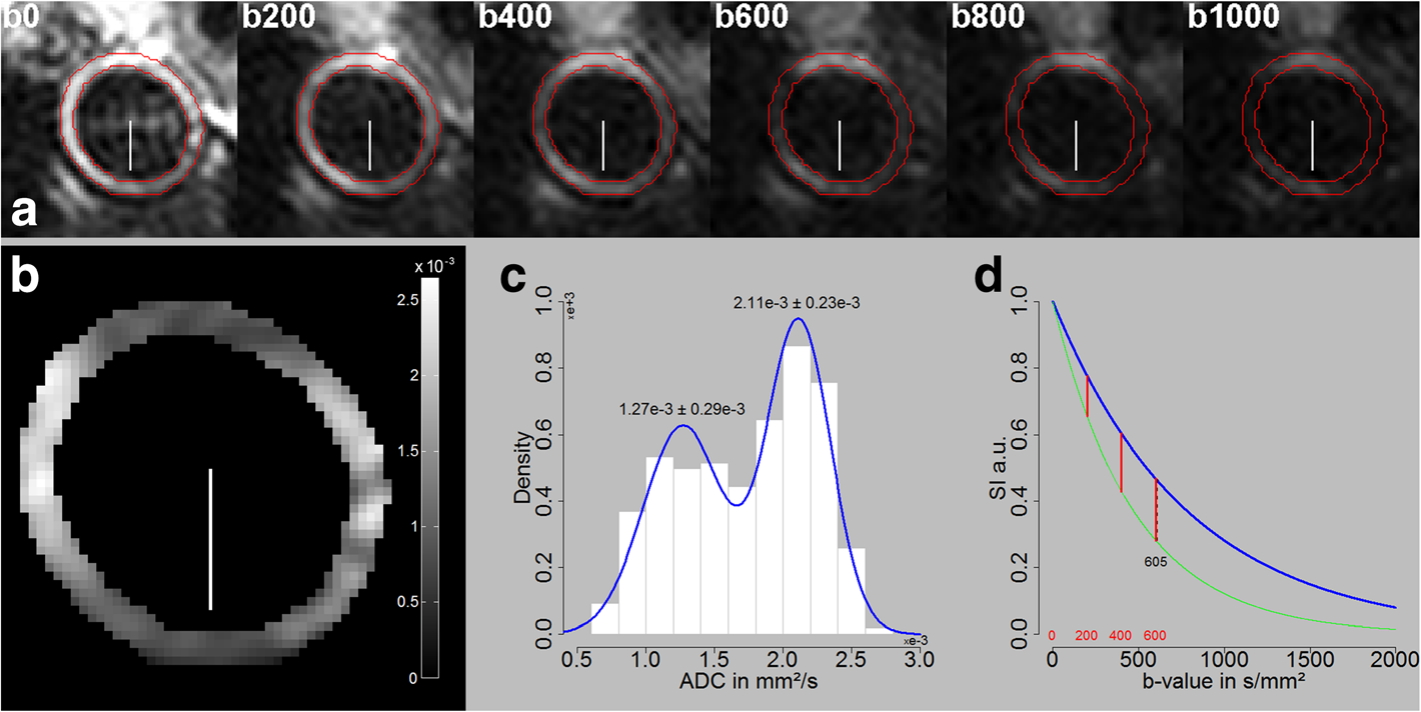
Vessel wall ADC map generated from six b-values images (0–1000 s/mm^2^). **a** B-value images along a certain gradient direction perpendicular to the slice direction (*white headless arrow*). **b** ADC map generated from the six b-value images illustrating a signal enhancement along and a signal drop perpendicular to the direction of the applied gradient direction. **c** Histogram distribution of ADC map indicating two populations of ADC values. **d** Result of signal simulation using equation **S**(**b**) = **S**_0_ **e** ^− **bD**^ and found ADC populations (*green line* 2.11 and *blue line* 1.27e-3 mm^2^/s). *Red vertical lines* indicate the final set of selected b-values and *black dashed vertical line* the maximum difference of the found ADC pool

To estimate optimal b-values and study the tissues’ anisotropic properties, an in vivo vessel wall ADC map was generated from multiple measurements with increasing b-values (0–1000 s/mm^2^) for a certain diffusion direction perpendicular to the slice direction (Fig. 3a). Given that a signal drop can be observed in the direction of the applied diffusion gradient direction and a signal enhancement perpendicular to it, a diffusion process perpendicular to the vessels’ longitudinal axis is assumable as exemplified in Fig. 3b. As shown in Fig. 3c, two pools of ADC values could be identified (mean values: 2.11e-3 ± 0.23e-3 and 1.27e-3 ± 0.29e-3 mm^2^/s). In addition, outlined in Fig. 3d is the final selected set of four equidistant b-value increments (0, 200, 400, 600 s/mm^2^) and the maximum difference of both populations found at a b-value of about 605 s/mm^2^. In addition to the diffusion decay factor *e* ^*(−bD)*^, signal loss is given by the vessel’s tissue T2 relaxation time and predefined echo time as expressed by the decay constant *e* ^*(−TE/T2)*^. All these effects contribute to a signal drop and justify a distribution of selected b-values between zero and the found maximum.

### FA, RD and primary eigenvalue vs. age relationship

**Fig. 4:**
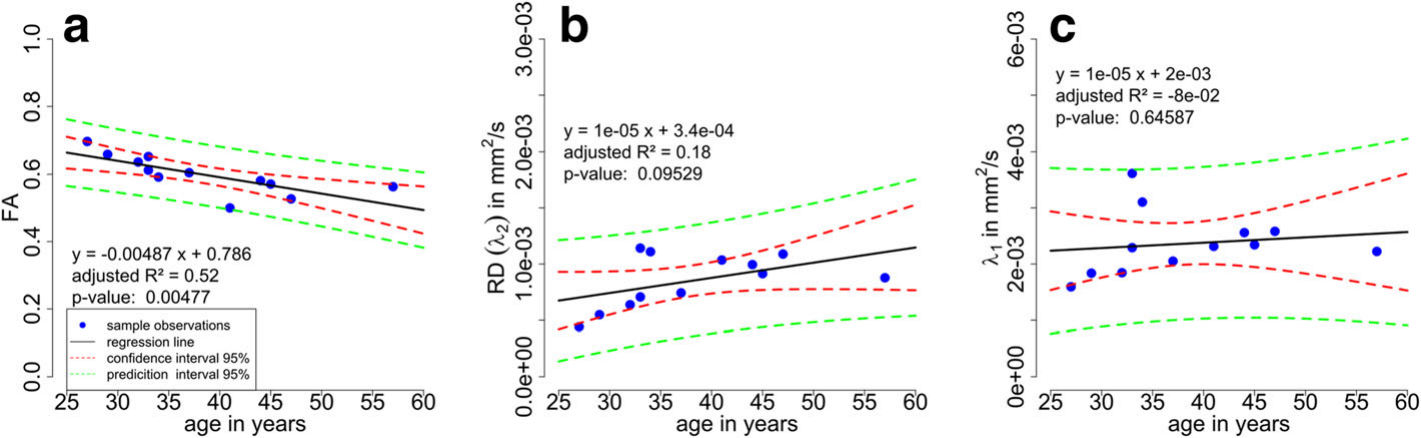
Linear regression analyses to compare FA, RD and λ_1_ vessel wall values with volunteer’s age. **a** Significant linear relationship (*p*-value = 0.00477) between mean FA and age; *black solid line* represents the fitted linear model (adjusted R^2^ = 0.52; linear equation: y = −0.0049x + 0.79). **b** Linear trend (*p*-value = 0.095) between mean RD and age; *black solid line* represents the fitted linear model (adjusted R^2^ = 0.18; linear equation: y = 1e-5x + 3.4e-4). **c** No linear relationship (*p*-value = 0.65) between the mean primary eigenvalues (λ_1_) and age was found; *black solid line* represents the fitted linear model (adjusted R^2^ = −0.08; linear equation: y = 1e-5x + 2e-3). In addition, calculated 95 % confidence (CI, *red dashed line*) and prediction intervals (PI, *green dashed line*) are plotted

In Fig. 4a, b and c, mean vessel wall FA, RD and primary eigenvalue (λ_1_) data for 12 male volunteers ranging from 0.7 for the youngest to 0.56 for the oldest are plotted as a function of age. The linear regression for the FA and age relationship was highly significant *P* < 0.01 with an adjusted R^2^ of 0.52 and the linear equation y = −0.0049x + 0.79. For the relationship between mean RD and age a linear trend *P* < 0.1 with an adjusted R^2^ of 0.18 was observed. No linear relationship *P* > 0.6 between the mean primary eigenvalues (λ_1_) and age was found. This analysis indicates that FA, measured within the vessel wall, is a sensitive parameter able to capture changes in the vascular architecture due to aging.

## Discussion

In this work, we demonstrated the feasibility of measuring diffusion anisotropy in human carotids in vivo in a non-destructive and non-invasive fashion. In contrast to previous works that aim for quantitative diffusion imaging in vivo (DW imaging, ADC values) along the slice direction (z-direction) our results suggests that tangential and radial diffusion is accessible by using a pulse sequence that enables high resolution DW imaging measurements in combination with a 2D gradient direction sampling-scheme orientated perpendicular to the vessel’s longitudinal axis. Furthermore, our in vivo and ex vivo results corroborate the fact that the principle diffusion direction is aligned perpendicular to the long axis of the artery as demonstrated by ex vivo studies on porcine aortas [9, 10]. Moreover, fiber orientation organizes more circumferential and coherent with increasing transmural pressure as was shown in an ex vivo investigation on human arteries [22]. Although our ex vivo setup was pressure free, the circumferential orientation of diffusion remained highly preserved (1.8 ± 7.2 °) another clear evidence for a dominant diffusion process perpendicular to the long axis of the vessel in vivo. Quantitative diffusion imaging along z-direction by evaluating the ADC may spare major information to ascertain arterial microstructure properties or changes in case of pathological tissue remodeling. Moreover, ADC depends on subject’s orientation relative to the magnetic field gradients introducing errors like scan-rescan variations or possible confound comparisons between individuals [23]. In contrast, DTI allows overcoming these issues by determining rotationally invariant parameters (e.g. eigenvalues λ) which values do not depend on subject’s orientation in the scanner reference frame. Hence, our framework implies that the combination of high resolution DW imaging with a special set of diffusion gradients aligned perpendicular to the long axis of the artery is essential to uncover important structural vessel wall information. Moreover, it was proofed that analyzing two-dimensional diffusion tensor directions is sufficient and also applicable in a clinical setup concerning the overall scan time. The excellent agreement of the tangential and radial components from the 2D and 3D-DTI analysis (Table 3) of the ex vivo porcine aorta further supports the reduction of the three-dimensional tensor to a two-dimensional one and the feasibility of our proposed method. Differences in the magnitude of mean FA values between 2D and 3D (Table 3) occur due to the missing third component (longitudinal diffusion). The reduction to a two-dimensional tensor causes an increase of the calculated FA values.

Recent investigations of ADC values along the z-direction (slice direction) were done on healthy volunteers and patients but the relevance of aging was not considered [6–8]. In contrast, our data reveal a highly significant relationship between FA vessel wall values and volunteer’s age. In detail, the data demonstrate a decrease in FA with advancing age indicating possible alterations of tissue microstructural integrity. Furthermore, RD values tend to increase with age indicating a possible decrease in cellularity and a conversion process to more extracellular space. In contrast, the primary eigenvalue λ_1_ indicate a rather constant behavior with advancing age. These indicators taken together may provide complex information about ongoing processes on the vascular architecture. However, the reported aging effect is notable but lifestyle may also influence vascular wall structure and was not covered by this proof of concept. To investigate this process more closely, further experiments are needed including more subjects. Another interesting application of the proposed method might be to study the influence of plaque components on the diffusion process. Future work on vessel wall diffusion imaging may help to understand the formation of such a complex disease.

To achieve accurate estimates of the self-diffusion tensor the sensitivity to motion-induced phase errors has to be reduced to a minimum. Motion is a severe factor in diffusion imaging in general and specifically when trying to access diffusion in the carotid wall [6, 8]. Several strategies used in this work directly address this problem. Firstly the suggested RESOLVE sequence inherently corrects for motion artifacts using a 2D navigator echo (correction for non-linear phase errors introduced by non-rigid body motion) [11, 24–26]. Secondly, pulse triggering accounts for the pulsatile motion of the arterial wall and thirdly, a co-registration procedure including translation followed b-spline based registration minimizes the distance of subsequent images. With this strategy no subject had to be excluded from the analysis due to motion. The repeatability of mean FA measurements (Table 2; CV: 2.5–5.4 %) clearly indicates the feasibility and robustness to evaluate the two-dimensional tensor in vivo.

Conventional single-shot EPI’s are fast sequences that enable the acquisition of a slice within a single repetition time but suffer from susceptibility artifacts and T2* blurring due to the prolonged time required to fill the k-space, low resolution and significant loss of SNR at higher resolution. Diffusion tensor imaging is inherently a technique suffering from low SNR. High spatial resolution cannot be achieved using such conventional ss-EPI due to the signal loss as a result of the prolonged echo time. Hence the use of rs-EPI is a prerequisite for high spatial resolution. It can be seen from the representative diffusion weighted images of Fig. 1 that the vessel wall can clearly be delineated and the signal is sufficient to calculate the diffusion tensor. The specific properties of the suggested RESOLVE sequence (short EPI echo spacing; 2D navigator based phase correction; parallel imaging using GRAPPA [27]) reduces the sensitivity to image distortions and motion induced artifacts and therefore make the sequence a well suited tool to analyze the two-dimensional tensor in vivo with sufficient SNR.

## Conclusions

In conclusion, we present a novel method to access tangential and radial diffusion of carotids in vivo by utilizing a pulse sequence that enables high resolution DW imaging in combination with a 2D diffusion gradient direction sampling scheme perpendicular to the longitudinal axis of the artery. In contrast to standard DW methods evaluating the ADC for a certain vascular segment along the artery’s axis our method allows to image the water diffusion tensor within the vessel wall with a high spatial resolution. Ex vivo studies on porcine arteries suggested a dominant circumferential fiber orientation rather than an organization in axial direction supporting our 2D approach. Furthermore, our results demonstrate a decrease in fractional anisotropy (FA) with advancing age indicating FA as a sensitive parameter able to capture changes in the vascular architecture with age. Moreover, analyzing 2D diffusion tensor directions is sufficient and applicable in a clinical setup concerning the overall scan time. Our data demonstrate for the first time the feasibility of measuring diffusion anisotropy in human carotids in vivo in a non-destructive and non-invasive fashion. Future work will investigate the influence of plaque components on the diffusion process, which may help to understand the formation of a complex disease.

## Abbreviations

2D: Two-dimensional
3D: Three-dimensional
ADC: Apparent diffusion coefficient
AS: Atherosclerosis
CMR: Cardiovascular magnetic resonance
CVDs: Cardiovascular diseases
DTI: Diffusion tensor imaging
DW: Diffusion weighted
FA: Fractional anisotropy
FLASH: Fast low angle shot
FOV: Field of view
GRAPPA: Generalized autocalibrating partially parallel acquisition
MDDW: Multi directional diffusion weighting
RD: Radial diffusivity
rs-EPI: Read-out segmented echo planar imaging
ss-EPI: Single-shot echo planar imaging
TE: Echo time
TI: Inversion time
TR: Repetition time
